# Scarring Alopecia With Coexisting Lichen Planus in a Child: A Rare Phenomenon

**DOI:** 10.7759/cureus.16730

**Published:** 2021-07-29

**Authors:** Muhammad Ammar B Hamid, Shahan Tariq

**Affiliations:** 1 Dermatology, National University of Medical Sciences, Rawalpindi, PAK

**Keywords:** alopecia, scarring, lichen planus, lichen planopilaris, hair loss

## Abstract

Lichen planopilaris (LPP) is characterized by chronic cicatricial alopecia that is lymphocytic in nature. The underlying pathophysiology of LPP is not well understood. Failure of therapeutic management occurs quite often resulting in significant psychosocial stress on the patient when they are unable to prevent further hair loss or reverse it. Although the occurrence of LPP is rare, it is especially seldom observed in the pediatric population. Herein, we discuss a case of a nine-year-old child who was recently diagnosed with LPP.

## Introduction

Patients of lichen planopilaris (LPP) usually present with polygonal patchy areas of alopecia. Inflammation in LPP leads to the destruction of hair follicles; therefore, the lesions are atrophic in nature [1]. These are accompanied by follicular papules having acicular hyperkeratosis and circumscribed erythema [2]. Clinically, LPP may appear quite similar to a number of other patchy hair disorders including central centrifugal cicatricial alopecia (CCCA), alopecia areata, traction alopecia, tinea capitis, and discoid lupus erythematosus (DLE) [3]. LPP is generally considered an irreversible condition. Treatment of LPP could be challenging considering its recalcitrant nature.

## Case presentation

A nine-year-old female presented to the dermatology department with complaints of hair loss, scaling, burning sensation, concomitant pain, and itching of the scalp. She also mentioned the recent development of purple lesions on her neck and back. The patient had visited a couple of general practitioners over the past year and her condition was interpreted as seborrheic dermatitis. She was treated with ketoconazole and selenium sulfide shampoo; however, her symptoms failed to improve. The onset of hair loss and purple-colored lesions eventually prompted a specialist referral. Based on the assessment of her parents, hair fall started approximately six months ago. It was gradual in onset and limited to the vertex of the scalp. There was no history of any endocrine dysfunction, autoimmune diseases, medications, use of cosmetics, or recent stressors. Even before the start of symptoms, the patient had been spending most of her time indoors with minimal exposure to direct sunlight. An account of a similar pattern of hair loss was not present in the family. According to the parents, the patient did not style her hair in a manner that would lead to traction or increased strain. She also denied self-pulling of hair or any urge to do so. Her dietary intake was adequate, and she had met all the developmental milestones timely.

**Figure 1:**
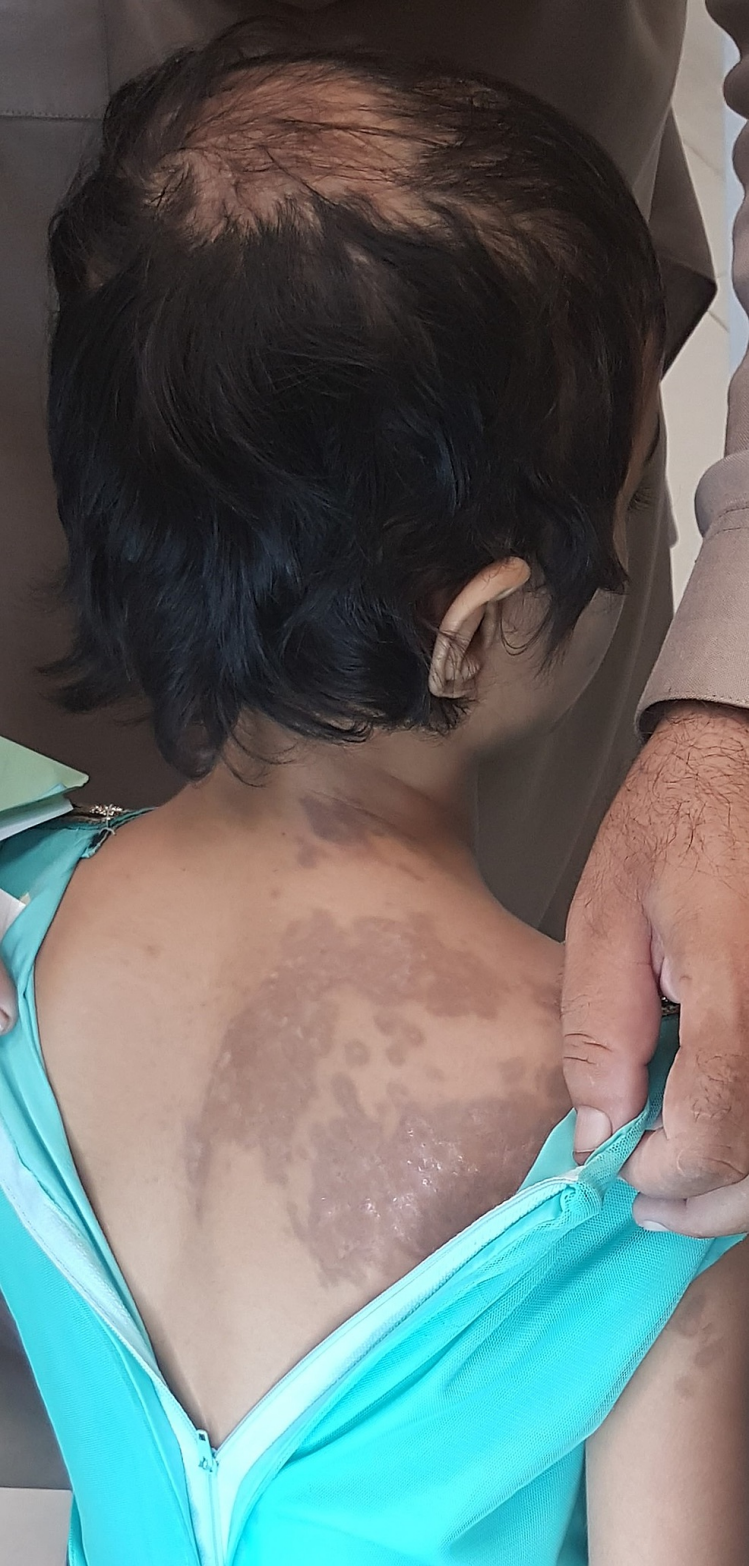
Picture demonstrating an area of scarring alopecia on the vertex and a lesion of lichen planus on the patient's back

On general examination, her vital signs were within normal range. There was no evidence of pallor, jaundice, lymphadenopathy, or nail changes. The thyroid was not enlarged and any evidence of systemic disease was not noticeable. On inspection of the scalp, a focal area of hair loss was evident on the vertex (Figure 1). It measured up to about 6 x 5 cm in size with irregular borders. Dermoscopy revealed perifollicular erythema along with loss of follicular openings due to scarring. Abnormal pigmentation was not observed. In addition, two dark purple-colored polygonal patches with irregular borders consistent with lichen planus (LP) were seen on the neck and back. Examination of the oral cavity was unremarkable. Since the suspicion for scarring alopecia was high, the patient underwent a punch biopsy for confirmatory diagnosis. A KOH prep test was negative. A number of investigations including complete blood count, liver function tests, renal function tests, thyroid-stimulating hormone, anti-nuclear antibody, and erythrocyte sedimentation rate were advised, all of which were within normal range. Hepatitis serology was also negative. The biopsy showed lymphocytic infiltration and fibroplasia of the perifollicular regions. Superficial scarring in the dermis was also detected. All of these findings were consistent with a diagnosis of LPP. The patient was thoroughly counseled regarding the disease and its prognosis. The patient was prescribed topical clobetasol (high-potency steroid), minoxidil, and an oral antihistamine. Due to her young age, she was advised to follow up monthly.

## Discussion

Clinically, LPP is categorized into three groups: classic LPP, frontal fibrosing alopecia, and Graham-Little-Piccardi-Lassueur syndrome [4]. Alopecia rarely occurs within the first decade of life; however, alopecia areata remains an exception, whereas pseudoalopecias along with congenital defects in the growth of hair are typically seen in the younger population. Children aged 10 years or less amount to approximately 5% of those consulting for problems in hair growth. Of these, around 60% have alopecia areata while 10% are affected by diffuse alopecia as well as trichotillomania. The remaining 20% of cases observed are quite infrequent [5]. One such rare condition is LPP in which the scalp is progressively affected, eventually resulting in scarring and development of a resistant form of hair loss [6]. 

The basis on which one disease is differentiated from another lies in obtaining a thorough history, carrying out a detailed clinical examination, and dermoscopy along with histopathology. Alopecia areata and tinea capitis are usually ruled out on examining the hair involved, which may be observed as a dystrophic/exclamation mark or a comma/corkscrew type of hair [7], respectively. Likewise, traction alopecia is largely due to traumatic hairstyling with a peripheral presentation. It not only involves the frontal and parietal areas but other regions of the scalp as well [8]. On the other hand, identical features such as fibrosis and follicular drop-out exist among all three diseases namely LPP, CCCA, and DLE. LPP presents with peripilar casts, which may also be observed in DLE. The most prominent features that are only observed in CCCA are typical peripilar white-gray halos. However, follicular plugs and red dots are absent in both LPP and CCCA [9]. Similarly, histopathology may reveal inflammatory infiltrate in CCCA. Although scarce, it is void of any involvement at the junctional interface between the dermis and epidermis compared to LPP where lichenoid infiltration involving the isthmus and infundibulum is its characteristic feature, along with the absence of sebaceous glands [10]. Moreover, distinct features such as Max Joseph spaces, interfollicular changes of LP, and a basal layer consisting of squamous cells distinguish LPP from the rest [11].

LPP is an uncommon find in the childhood population and is often characterized by clinical features not seen in the classical disease. LP was studied in the children population of the northern areas of the subcontinent for over a period of more than 12 years where it was found that LPP made up only a small fraction of the rarer forms of LP [12]. Only a very small number of such cases have been observed worldwide to this date. A retrospective review from the year 1976 to 2013 was conducted. It comprised patients under the age of 18 years who were analyzed to further define the clinical and pathologic features of pediatric LPP. Four pediatric LPP patients, ranging from 13 to 16 years of age, consisting of three males and one female, were identified [13]. The case we observed was an extraordinarily unique find, where a child under the age of 10 years was found to be suffering from LPP.

Corticosteroids play a major role in the management of LPP as first-line therapy. Mycophenolate mofetil is generally a safe medication that is well tolerated and can be opted as the second-line regimen for the treatment of LPP following the failure of corticosteroids, either intralesional or topical [14]. A study involving children observed that the management/response to treatment of LPP is similar to that in adults [15]. Keeping in view the safety profile, topical steroids remain the most suitable option for children; however, more rigorous monitoring is required for systemic therapy.

## Conclusions

LPP in the pediatric population is seen very rarely. The extremely recalcitrant nature of this disease makes the treatment challenging. There still exist a number of inconsistencies in treatment efficacy concerning LPP due to difficulty in comparison between trials. Hence, we present forth our case in order to add to the database of existing features and characteristics of LPP. Randomized controlled double-blinded studies on this subject have become the need of the hour so as to advance the literature on the treatment modalities of LPP.
